# Characterization of ****β****-Glucosidase Produced by *Aspergillus niger* under Solid-State Fermentation and Partially Purified Using MANAE-Agarose

**DOI:** 10.1155/2014/317092

**Published:** 2014-04-01

**Authors:** Anderson Baraldo Junior, Diogo G. Borges, Paulo W. Tardioli, Cristiane S. Farinas

**Affiliations:** ^1^Departamento de Engenharia Química, Universidade Federal de São Carlos, Rodovia Washington Luiz km 235, 13565-905 São Carlos, SP, Brazil; ^2^Embrapa Instrumentação, Rua XV de Novembro 1452, 13560-970 São Carlos, São Paulo, SP, Brazil

## Abstract

**β**-Glucosidase (BGL) is a hydrolytic enzyme with specificity for a wide variety of glycoside substrates, being an enzyme with a large range of biotechnological applications. However, enzyme properties can be different depending both on the microorganism and the cultivation procedure employed. Therefore, in order to explore potential biocatalytical applications of novel enzymes, their characterization is essential. In this work, a BGL synthesized by a selected strain of *Aspergillus niger* cultivated under solid-state fermentation (SSF) was partially purified and fully characterized in terms of optimum pH, temperature, and thermostability. The single-step purification using MANAE-agarose in a chromatographic column yielded an enzyme solution with specific activity (17.1 IU/mg protein) adequate for the characterization procedures. Electrophoresis SDS-PAGE and size-exclusion chromatography analysis resulted in an estimated molecular mass of 60 kDa. Higher enzyme activities were found in the range between 40 and 65°C and between pH 4 and 5.5, indicating an interesting characteristic for application in the hydrolysis of lignocellulosic biomass for biofuels production. Thermostability studies of purified BGL resulted in half-lives at 37°C of 56.3 h and at 50°C of 5.4 h. These results provide support for further studies of this enzyme towards revealing its potential biotechnological applications.

## 1. Introduction

The bioconversion of lignocellulosic materials into fermentable sugars requires a multienzymatic system containing cellulases, xylanases, and other accessory enzymes [1]. This enzymatic cocktail is one of the most costly inputs affecting the economic viability of the biochemical route for biomass conversion into biofuels and other chemicals [2]. In this process, cellulases act synergistically in the hydrolysis of cellulose: endoglucanases and exoglucanases or cellobiohydrolases (CBHs) act directly on the cellulose fiber, while *β*-glucosidase (BGL) hydrolyzes oligosaccharides and cellobiose into glucose [3]. The adequate proportion of each of these enzymes is crucial for an efficient hydrolysis, since the end product of CBH activity, which is cellobiose, is a product inhibitor for these enzymes [4–6]. Thus, BGL activity is important towards reducing inhibition effects. More importantly, cellobiose needs to be further degraded into the simple sugar glucose before it can be utilized by conventional yeasts for ethanol fermentation [7].

Most commercial cellulases are produced by filamentous fungi of the genera* Trichoderma* and* Aspergillus* [8]. However, the amount of BGL secreted by* Trichoderma *is not sufficient for an efficient biomass conversion [5], since the extent of cellulose conversion into glucose is dependent upon the amount of active BGL enzymes. On the other hand,* Aspergillus* strains, such as* Aspergillus niger,* has been used for BGL production that is often used as a supplementation of commercial enzymatic cocktails [4, 5]. Besides,* A. niger* is prized in the industry for its high fermentation capacity, elevated levels of proteins secreted, and the variety of enzymes produced for diverse applications, including a combination of enzymes for degradation of plant cell wall polysaccharides [9, 10]. Thus, production, purification, and characterization of* A. niger *BGL enzymes arise as important steps throughout this application.

The purification and characterization of BGL produced from different sources have been previously described in the literature using a range of different techniques [4, 11–18]. Nevertheless, most of the purification procedures include a sequence of steps that often results in a reduced recovery value or even some unwanted modification in the enzyme that can lead to activity loss. In order to preserve enzymatic activity it is necessary to reduce the purification steps and to produce the enzymes in a more concentrated medium. In this sense, the use of solid-state fermentation (SSF) is particularly advantageous for enzyme production, since the enzymes are produced in a more concentrated form when compared to submerged fermentation [19]. Given the potential of* A. niger* for BGL production there is a great interest in the development of efficient purification protocols and its characterization.

Here, we studied the purification and characterization of BGL produced by a selected strain of the filamentous fungi* A. niger* cultivated under solid-state fermentation. The purification of BGL from the enzymatic extract was initially evaluated using two different adsorbents (MANAE-agarose and Glucose-agarose) under batch experiments. Purification was further studied using a chromatographic column with MANAE-agarose. The partially purified *β*-glucosidase was thoroughly characterized in terms of the variables pH and temperature by using response surface methodology. The enzyme thermostabilities at 37 and 50°C were also evaluated.

## 2. Materials and Methods

### 2.1. Microorganism

The microorganism used in this study was the wild-type strain* A. niger* 12 from the Embrapa Food Technology collection (Rio de Janeiro, Brazil). Microorganism activation was carried out in basic medium agar slants incubated for 7 days at 32°C [20]. After this period, conidia were harvested by adding 10 mL of 0.1% Tween-80 to the slant.

### 2.2. SSF Cultivation Conditions

Solid-state fermentation cultivations were carried out as described in [21]. Briefly, 500 mL Erlenmeyer flasks containing 10 g of wheat bran with a moisture level of 60% (adjusted with 0.9% (w : v) ammonium sulfate solution in 0.1 mol/L HCl) were sterilized and then inoculated with a suspension of 10^7^ spores per gram of solid medium. Cultivations were carried out at 32°C for 72 hours. After this period, the enzymes were extracted by adding 0.2 mol/L acetate buffer at pH 4.5. The samples were stirred at 120 rpm for 1 h and the enzymatic solution was recovered by filtration. The recovered enzyme extracts were stored at −18°C for further analysis.

### 2.3. *β*-Glucosidase Partial Purification Using MANAE-Agarose

Monoaminoethyl-N-aminoethyl (MANAE) agarose gel was prepared as described by [22] using glyoxal supports which were prepared according to [23]. For preparation of MANAE-agarose, to the support prepared from glyoxal-agarose, 200 mL of 2 M ethylenediamine (EDA) solution at pH 10 was added to a 35 g of glyoxal-agarose support. After 2 h of gentle agitation, sodium borohydride was added to a final concentration of 10 mg/mL. Again, after 2 h of gentle agitation, the MANAE-agarose support was washed successively with 100 mM acetate buffer pH 4, 100 mM borate buffer pH 9 and finally distilled water.

Purification of BGL present in the crude enzymatic extract was initially evaluated using batch experiments undertaken in 50 mL flasks. For this, the MANAE-agarose gel previously described was added to the enzymatic solution at a 1 : 2 (m/v) ratio. This suspension was kept under gently agitation at 25°C. Total protein and enzymatic activity were periodically measured in the supernatant. After the period considered sufficient for equilibrium to be achieved, the MANAE-agarose gel was filtered and washed with a 5 mM phosphate buffer at pH 7.0. The adsorbed enzyme was released from the support by incubation with a gradient of 150 to 600 mM NaCl solution at pH 7.0.

For the chromatographic assay, a 25 mL column was packed with 5 g of the adsorbent MANAE-agarose and equilibrated with the equilibration buffer (5 mM phosphate buffer at pH 7.0). Enzymatic extract (3 mL) was fed into the column, followed by a washing step with this same equilibration buffer. Elution was undertaken with a NaCl step gradient using the equilibration buffer containing 150 to 600 mM NaCl. The flow rate during all procedures was 0.4 mL/min and the 3.0 mL fractions collected were analyzed for total protein and BGL activity.

### 2.4. *β*-Glucosidase Partial Purification Using Glucose-Agarose

Sepharose 6B-CL, acquired from Sigma (St. Louis, MO), was used as support for the biospecific affinity purification of BGL. The affinity support was derivatized by activation with bisoxirane following the protocol described by [24] and coupling the biospecific ligand glucose. Briefly, for the immobilization of the ligand glucose, the activated gel was washed with distilled water to remove excess dioxirane and stabilized for 5 minutes in a solution of NaOH 0.1 M. For each gram of activated and dried gel, 2 mL of 0.1 M NaOH containing 20% (w/v) glucose was added and stirred for 16 hours at 45°C. After immobilization of the ligand, the gel was washed with distilled water and sodium phosphate buffer 50 mM, pH 7, in order to eliminate unfixed glucose molecules. The gel was then stored at 4°C in the same buffer containing 0.1% (w/v) sodium benzoate until use.

For the purification procedure, a suspension containing the BGL enzymatic complex and the affinity support (Agarose-Glucose) at a 1 : 2 (m/v) ratio was prepared. This suspension was maintained under gentle stirring for a time suitable for the maximum adsorption of the enzymes. Protein concentration and enzyme activity were monitored regularly in the supernatant. When the protein concentration in the supernatant and/or enzymatic activity became constant, the gel was filtered, washed with sodium phosphate buffer 5 mM, pH 7, and resuspended into a small volume of the same buffer. For the elution step, the gel was resuspended with a supersaturated solution of glucose (maximum of 1 mol/L glucose) and was left under mild stirring for 30 min. The glucose concentration of the medium was gradually increased until the protein concentration of the supernatant was equal to or near the protein concentration of the total suspension. At this point, it could be inferred that the maximum amount of the enzyme attached to the affinity support was eluted. However, with this process the enzyme is obtained and purified BGL eluted in a solution containing high concentrations of glucose (1 mol/L glucose). As glucose can act as an inhibitor in the reaction catalyzed by BG, a filtration process was made to remove this glucose solution. The solution was placed into a 10 kDa ultrafiltration system and centrifuged at 10.000 g at 4°C, so that the glucose could pass through the filter, while the protein fraction remained. In the final solution without glucose, BGL activity and total protein were measured and the purification factor was calculated.

### 2.5. Determination of Optimum pH and Temperature

A full factorial design followed by response surface analysis was used to evaluate the effect of temperature and pH on BGL activity. Optimum pH and temperature for BGL were determined by assaying the corresponding activity at different temperatures (from 23 to 87°C) and pH values (from 2.4 to 6.6), selected according to the experimental design conditions. Cellulase enzymes usually have optimum activity at pH around 5.0 and temperature of 50°C. Therefore, pH and temperature values around these ranges were selected. The experimental design selected was a central composite design comprising eleven runs, corresponding to four cube points, four axial points, and three central points, with the experiments carried out in a random order. The dependent variable (response) was BGL activity. The Statistica software (Statsoft, version 7. 0) was used to analyze the experimental data, the generation of the ANOVA (analysis of variance) data, and the plotting of response surfaces.

A second-order polynomial model of the form of (1) was used to fit the data:

(1) $$\begin{array}{c} Y=\beta_{0}+\beta_{1}X_{1}+\beta_{2}X_{2}+\beta_{11}X_{1}^{2}+\beta_{22}X_{2}^{2}+\beta_{12}X_{1}X_{2}, \end{array}$$

where *Y* is the predicted response variable; *β*_0_ is the intercept term; *β*_1_ and *β*_2_ are the linear coefficients; *β*_11_ and *β*_22_ are the squared coefficients; *β*_12_ is the interaction coefficients; and *X*_1_ and *X*_2_ are the coded independent variables representing the pH and the temperature, respectively.

### 2.6. Enzyme Thermostability

BGL thermostability at temperatures of 37 and 50°C was evaluated by measuring the residual enzyme activity after each 24 h during a 96 h total incubation period in the absence of substrate. The enzyme's half-lives were calculated according to [30] using the single-step nonfirst-order model proposed by [31] to fit the experimental data. The activity time expression used (see (2)) relates the activity (*a*) to the parameter *k*_1_, the first-order deactivation rate constant, the parameter *α*_1_, and the long-term level of activity:

(2) $$\begin{array}{c} a=\left(1-\alpha_{1}\right)\exp\left(-k_{1}\cdot t\right)+\alpha_{1}.\; \end{array}$$

The two-parameter model was fitted to the residual activity data using the Levenberg-Marquardt method of iterative convergence, at 0.95 confidence level.

### 2.7. Analytical Measurements

BGL activity was determined by incubating a mixture of 1 mL of the appropriately diluted enzymatic extract and 1 mL of a 0.015 mol/L solution of cellobiose (Sigma, USA) prepared in 0.05 mol/L sodium citrate buffer pH 4.8 as substrate for 30 min at 50°C. The reaction was stopped by submersion in boiling water for 5 min. The quantification of the glucose released was performed by using an enzymatic kit for glucose measurement (Laborlab, Brazil). Total protein was measured using the Bradford method [32]. Bovine serum albumin was used as standard. The protein molecular mass profiles were evaluated by SDS-PAGE electrophoresis conducted under denaturing conditions as described by [33]. Gels (15%) were stained with Coomassie Blue. Size exclusion chromatography in a Superdex 200 100/300 GL (GE, USA) column was also used for molecular mass evaluation. The enzyme was eluted with 50 mM phosphate buffer (pH 7.0) containing 15 mM NaCl at a flow rate of 0.5 mL/min.

## 3. Results and Discussion

### 3.1. BGL Production and Partial Purification

**Figure 1:**
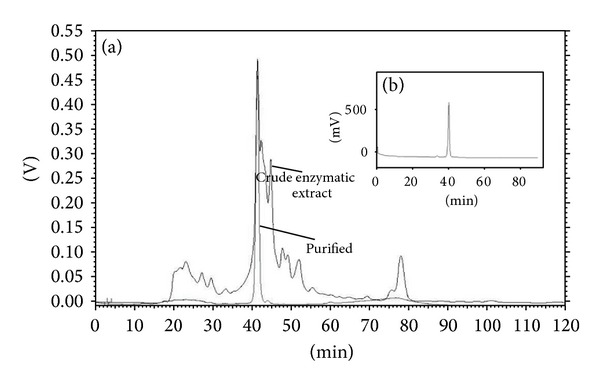
HPLC size-exclusion chromatograph of enzymatic extract before and after purification using MANAE-agarose (a) and glucose-agarose (b) supports in batch experiments.

The purification of BGL present in the cellulolytic enzyme complex produced by the filamentous fungus* A. niger* grown on wheat bran under SSF was initially evaluated by carrying out batch experiments for both MANAE-agarose and glucose-agarose supports (Table 1). The purification factor using MANAE-agarose was 2-folds higher than the one using Glucose-agarose. Even though the purification factors achieved using the two adsorbents were different, the analysis of both purified materials by size exclusion on a high resolution size exclusion column resulted in a single peak (Figure 1), demonstrating their effectiveness for BGL purification.

**Figure 2:**
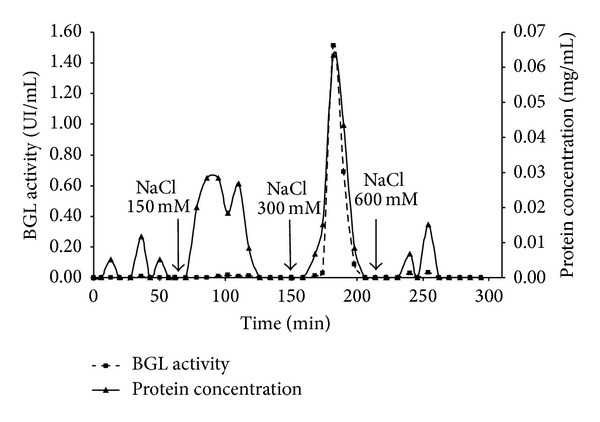
MANAE-agarose chromatogram for *β*-glucosidase purification. Feeding step: enzymatic extract produced by* A. niger* under SSF. Elution was carried out using a NaCl step gradient with 5 mM citrate buffer pH 7.0.

Based on these results, the support MANAE-agarose was selected for further studies of purification under dynamic conditions using a chromatographic column.  Figure 2 shows the chromatogram for BGL purification using a MANAE-agarose loaded column and Table 2 shows the mass balance of BGL purification for the respective system. It can be observed that a considerable amount of total proteins and BGL was not adsorbed on the MANAE-agarose gel as it passed through the column during the feed and washing steps. Using a buffer solution of low ionic strength (50 mM NaCl), few proteins were desorbed and almost no BGL activity was presented in the elution samples. With a buffer solution of 150 mM NaCl, the desorption process showed an interesting result. It could be observed that, although a considerable amount of total proteins was desorbed, no BGL activity was shown in the samples, thereby indicating that at this ionic strength only minor proteins and contaminants are desorbed from the support. BGL was desorbed from MANAE-agarose by using a buffer of ionic strength of 300 mM. These samples were analyzed presenting a high BGL activity and total protein desorbed compared to the other ionic strengths buffers.

**Figure 3:**
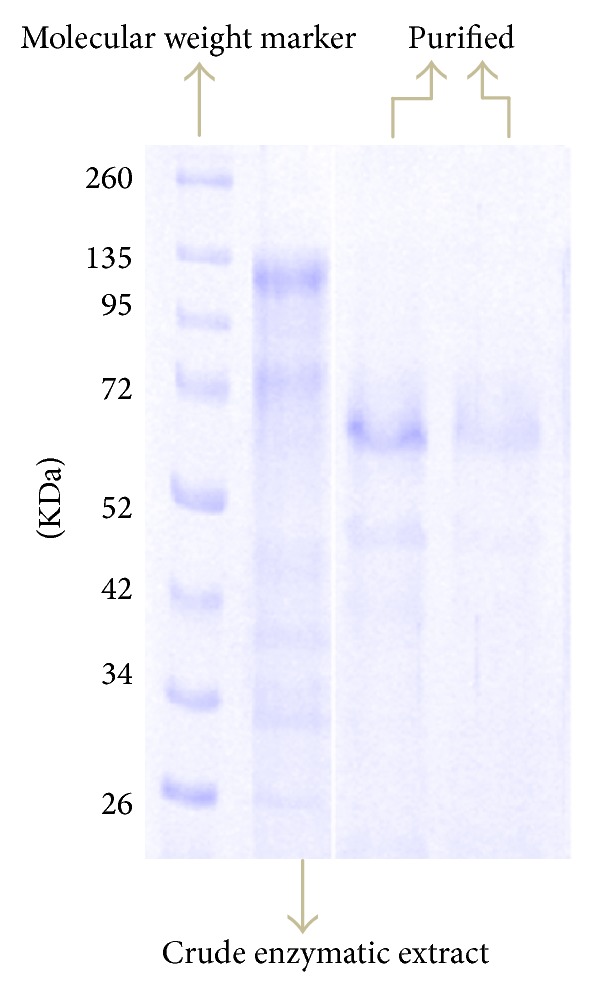
Electrophoresis profile of enzymatic extract after purification using MANAE-agarose packed column.

By using a MANAE-agarose chromatographic column for BGL purification, specific activity increases from 11.2 IU/mg in the crude enzymatic extract to 17.1 IU/mg, considering the elution using 300 mM NaCl. This corresponds to a purification factor close to 2. Nevertheless, the effectiveness of BGL purification under this condition can be verified in the SDS-PAGE electrophoresis gel (Figure 3). Analysis of the enzyme by SDS-PAGE revealed a major band with an estimated molecular mass of 60 kDa. Size exclusion chromatography on a Superdex 200 high resolution column resulted in the elution of BGL as a peak also between 50 and 70 kDa (data not shown). This result indicates that BGL may be present as a monomer in solution. This result is in agreement with the theoretical molecular mass of BGL (close to 75 kDa), according to [4].

### 3.2. Characterization of BGL from* A. niger*

The physicochemical characterization of enzymes is important in order to identify the performance of the enzyme under different work conditions. However, enzyme properties can be different depending on the microorganism producing them. Therefore, in order to explore potential biocatalytical applications of enzymes produced by different strains, the characterization of these enzymes is essential. Some of the enzyme properties considered important for production, purification, and application are its size/molecular weight, optimal pH, and temperature for enzyme activity and stability. Described in the following sections is the characterization of a MANAE-agarose partially purified BGL produced by a strain of* Aspergillus niger* cultivated under solid-state fermentation. Our previous results of BGL properties present in a crude enzymatic extract were used for comparison [21].

#### 3.2.1. Effects of pH and Temperature on BGL Activity

Initially, the effects of pH and temperature on BGL activity were evaluated as this characterization is important for selection of process conditions. The active sites on enzymes are frequently composed of ionizable groups which must be in the proper ionic form to maintain the conformation of the active site, bind the substrate, or catalyze the reaction. In terms of the temperature effect on enzymes activity, it is expected a faster velocity as the temperature rises, as an increase in temperature imparts more kinetic energy to the molecules resulting in more productive collisions per unit of time. However, if the molecule absorbs too much energy, the enzyme can be denatured and lose its catalytic activity [34].

**Figure 4:**
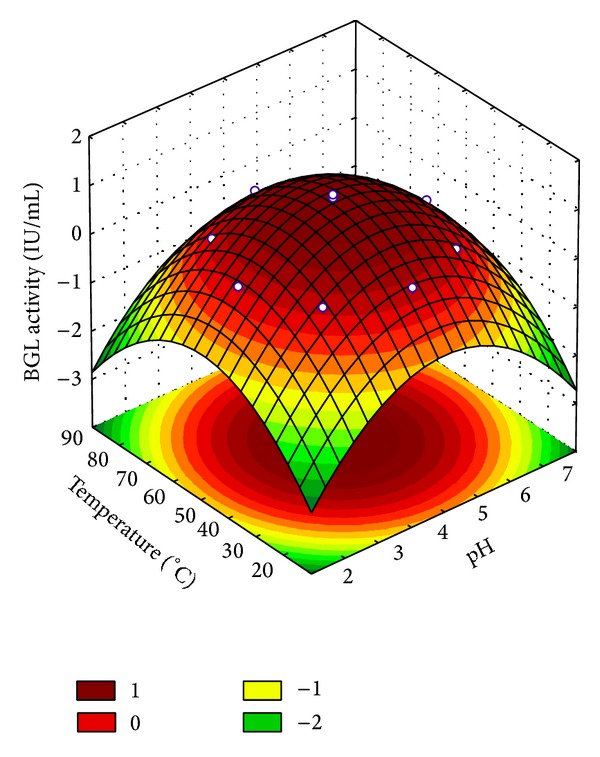
Surface response showing the pH and temperature effect on *β*-glucosidase activity partially purified using MANAE-agarose packed column.

Here, the pH and temperature effects on a partially purified BGL were evaluated by using the statistical design of experiments and response surface methodology analysis.  Table 3 presents the results of the complete factorial design for BGL activities under the different conditions evaluated.  Table 4 exhibits the coefficients of the mathematical model and statistical parameters. The quadratic effects of both pH and temperature on BGL activity were significant at 95% confidence limit (*P*-value < 0.05). Both variables showed a negative effect on BGL activity. The ANOVA analysis for BGL activity showed that the coefficient of correlation (0.9923) and the *F* test (115.43 times higher than the listed *F* value at 95% level of confidence, resp.) were very satisfactory for the prediction of the model used to describe the response surface plot of the enzyme activities as a function of pH and temperature (Figure 4).

The data analysis allowed defining an optimum range of temperature and pH for higher enzyme activity, as well as the degree of significance of each variable. The statistical analysis revealed that the optimum values of temperature for partially purified BGL were found to be in the range between 40°C and 65°C, and the optimum pH range was found between 4 and 5.5. Higher experimental values of enzymatic activity were found at the condition of the central point, which is at pH 4.5 and 55°C. This optimum range is in agreement with the results obtained for the BGL present in the crude enzymatic extract, as previously described in [21].

Several studies focusing on the characterization of BGL in terms of pH and temperature parameters have been made due to a large interest in understanding the action of this enzyme under different conditions. The optimum pH and temperature of BGL from different sources are described in Table 5. The optimum pH ranges from 3.6 to 5.0, while the optimum temperature ranges from 55 to 70°C. However, most of these studies describe the evaluation of one parameter at a time. Finding the optimal pH and temperature includes a study of both variables at the same time. In this context, the use of statistical experimental design followed by response surface methodology (RSM) to identify the optimal values of pH and temperature can be very effective to analyze the relationships among these parameters. The conventional method for optimizing a multivariable system analyzing one factor at a time does not access the interaction effects between the variables. This interaction can result in a synergistic effect, that is, a pH and temperature condition in which the enzymes have a higher activity value. Another advantage of using the statistical methodology was the definition of an optimum temperature and pH range, rather than a specific value, allowing more flexibility during process development.

The optimum range of temperature for partially purified BGL, between 40 and 65°C, and the optimum pH range, between 4 and 5.5, can be very interesting characteristics for application in the hydrolysis of lignocellulosic materials. For the simultaneous saccharification and fermentation process configuration, a compromise has to be found, since the optimal temperature for enzymatic hydrolysis is usually higher (around 55°C when using fungal enzymes) than that of fermentation by yeasts (around 30°C) [35]. Therefore, an enzyme with this relatively broad range of optimum temperature would be of interest.

#### 3.2.2. BGL Thermostability

Evaluation of enzyme thermostability is very important towards defining the optima range of process conditions in order to preserve the catalytic activity during the hydrolysis. Besides, advantages such as faster reaction rates, lower viscosity, and increased solubility of the substrate have been proposed for the use of thermostable enzymes in biotechnological processes [36]. Thermostable enzymes in the hydrolysis of lignocellulosic materials can also have several potential advantages: higher specific activity (decreasing the amount of enzyme needed), higher stability (allowing elongated hydrolysis times), and increased flexibility for the process configurations [37].

**Figure 5:**
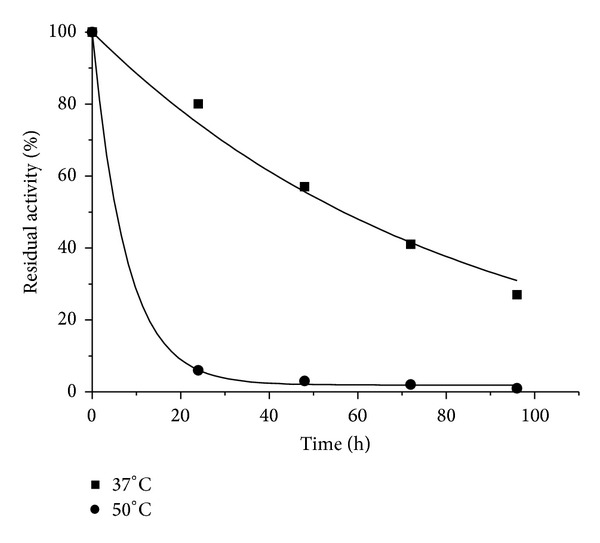
Thermostability at 37 (●) and 50°C (■) of *β*-glucosidase activity partially purified using MANAE-agarose packed column.

Thermostability of partially purified BGL at 37°C and 50°C was evaluated (Figure 5). These two temperatures were selected considering that the use of 37°C is an intermediate condition for a simultaneous saccharification and fermentation process configuration, since the yeast usually has an optimal temperature around 30°C and enzymes around 50°C. The model proposed by [31] was used to fit the experimental data and the enzyme's half-lives were then calculated using the fitted model according to [30].  Table 6 shows the results of the model parameters *k*_1_ and *α*_1_ and the half-life for BGL activity present in the crude enzyme extract and after the purification step. A higher stability at 37°C and 50°C was observed for BGL present in the crude extract in comparison to the partially purified enzyme (Table 6), showing that the purification procedure negatively influenced BGL thermostability. This result was rather unexpected since the purification procedure usually favors enzyme stability due to the removal of proteases and other proteins that could form aggregates with the molecule of interest.

A reduction in BGL stability after purification found in our results may be related to the removal of some stabilizing compound originally present in the medium. Nevertheless, it is important to note that this thermostability analysis is carried out exclusively for characterization purpose. In practice, different conditions such as the presence of the substrate and stabilizing agents can circumvent this characteristic.

## 4. Conclusion

The purification protocol used to purify BGL from the enzymatic extract obtained by the cultivation of* Aspergillus niger* under SSF was very efficient, since by using a MANAE-agarose adsorbent it was possible to obtain a partially purified enzyme in a single purification step. To better understand the characteristics of this enzyme and extend the knowledge about its potential applications, a complete characterization in terms of optimum pH and temperature and thermostability was performed. The methodology employed here was very effective in estimating enzyme behavior under different pH and temperature conditions. Higher enzyme activities were found to be in the range between 40 and 65°C and between pH 4 and 5.5. In terms of thermal stability, the enzymatic extract was found to be highly stable at both temperatures tested 37 and 50°C when present in the crude enzymatic extract, although the purification procedure showed a negative effect on BGL thermostability.

## Figures and Tables

**Table 1:** Mass balance of *β*-glucosidase purification using MANAE-agarose and glucose-agarose supports in batch experiments.

Adsorbent	BGL feed (IU)	BGL adsorbed (IU)	Total protein feed (mg)	Total protein adsorbed (mg)	Initial specific activity (IU/mg)	Final specific activity (IU/mg)	Purification factor
MANAE-agarose	78.6	27.7	10.2	2.1	7.7	20.1	2.6
Glucose-agarose	44.9	12.3	7.8	2.8	5.8	7.5	1.3

**Table 2:** Mass balance of *β*-glucosidase purification using MANAE-agarose support in a chromatographic column.

	MANAE-agarose			
	Total protein		*β*-Glucosidase	
	mg	%	IU	%
Feeding	2.95	100	33.08	100

Washed out	1.96	66.4	25.08	75.8
Elution NaCl (mM)				
50	0.07	2.4	0.03	0.1
150	0.39	13.2	0.14	0.4
300	0.41	13.9	7.00	21.2
600	0.07	2.4	0.20	0.6
Total recovery	**2.90**	**98.3**	**32.5**	**98.2**

**Table 3:** Experimental conditions and results of the statistical experimental design for *β*-glucosidase activity.

Trial	*T* (°C)	pH	*β*-Glucosidase (IU/mL)
1	80 (1)	6 (1)	0.028
2	80 (1)	3 (−1)	0.027
3	30 (−1)	6 (1)	0.056
4	30 (−1)	3 (−1)	0.071
5	87 (1.41)	4.5 (0)	0.092
6	23 (−1.41)	4.5 (0)	0.175
7	55 (0)	6.6 (1.41)	0.084
8	55 (0)	2.4 (−1.41)	0.028
9	55 (0)	4.5 (0)	0.971
10	55 (0)	4.5 (0)	1.059
11	55 (0)	4.5 (0)	1.034

**Table 4:** Coefficient values and statistical analysis for *β*-glucosidase activity.

	*β*-Glucosidase	
	Coefficients	*P* value
Mean*	1.021	0.0000
*T *	−0.024	0.2003
*T* ^²^*	−0.456	0.0000
pH	0.008	0.6333
pH^²^*	−0.495	0.0000
*T*·pH	0.004	0.8670

*R *	0.9923	
*F* value	514.81	
*F*_cal_/*F*_listed_	115.43	

*Significant at 0.05 level; *R* = coefficient of determination.

**Table 5:** Literature values for optimum pH and temperature of different BGL.

Microorganism source	Optimum pH	Optimum temperature (°C)	Reference
*Penicillium pinophilum *	5.0	60	[12]
*Penicillium funiculosum *	4.0 and 5.0	60	[25]
*Aspergillus glaucus *	3.6	60	[16]
*Aspergillus terreus *	5.0	60	[26]
*Aspergillus niger *	4.0	60	[27]
*Aspergillus niger *	4.5	60–70	[6]
*Aspergillus niger *	4.5	55	[18]
*Aspergillus niger *	4.5	60	[13]
*Aspergillus niger *	4.5–5.0	55–60	[28]
*Aspergillus niger *	5.0	55	[29]
*Aspergillus niger *	4.0–5.5	40–65	This work

**Table 6:** Half-life values for BGL under different temperatures.

	BGL in the crude extract [21]		Partially purified BGL	
Temperature (°C)	**37**	**50**	**37**	**50**
*α*_1_ (h^−1^)	0.8408	0	0	0.0191
*k*_1_	0.0327	0.0047	0.0122	0.1316
Half-life (h)*	341.5	148.1	56.3	5.4

*Half-lives calculated from the model equation.
